# Tumor necrosis factor receptor superfamily 10B (TNFRSF10B): an insight from structure modeling to virtual screening for designing drug against head and neck cancer

**DOI:** 10.1186/1742-4682-10-38

**Published:** 2013-06-01

**Authors:** Rana Adnan Tahir, Sheikh Arslan Sehgal, Naureen Aslam Khattak, Jabar Zaman Khan Khattak, Asif Mir

**Affiliations:** 1 Department of Bioinformatics and Biotechnology, International Islamic University, Islamabad, Pakistan; 2 National Center for Bioinformatics, Quaid-i-Azam University, Islamabad, Pakistan; 3 Department of Biochemistry, PMAS Arid Agriculture University, Rawalpindi, Pakistan

**Keywords:** Head and neck cancer, Modeling, Tumor necrosis factor, TNFRSF10B, Docking, MODELLER, Phylogenetic, Virtual screening, Inhibitors, Bioinformatics

## Abstract

**Background:**

Head and neck cancer (HNC) belongs to a group of heterogeneous disease with distinct patterns of behavior and presentation. TNFRSF10B, a tumor suppressor gene mapped on chromosome 8. Mutation in candidate gene is responsible for the loss of chromosome p arm which is frequently observed in head and neck tumors. TNFRSF10B inhibits tumor formation through apoptosis but deregulation encourages metastasis, migration and invasion of tumor cell tissues.

**Results:**

Structural modeling was performed by employing MODELLER (9v10). A suitable template [2ZB9] was retrieved from protein databank with query coverage and sequence identity of 84% and 30% respectively. Predicted Model evaluation form Rampage revealed 93.2% residues in favoured region, 5.7% in allowed region while only 1 residue is in outlier region. ERRAT and ProSA demonstrated 51.85% overall quality with a −1.08 Z-score of predicted model. Molecular Evolutionary Genetics Analysis (MEGA 5) tool was executed to infer an evolutionary history of TNFRSF10B candidate gene. Orthologs and paralogs [TNFRSF10A & TNFRSF10D] protein sequences of TNFRSF10B gene were retrieved for developed ancestral relationship. Topology of tree presenting TNFRSF10A gene considered as outgroup. Human and gorilla shared more than 90% similarities with conserved amino acid sequence. Virtual screening approach was appliedfor identification of novel inhibitors. Library (Mcule) was screened for novel inhibitors and utilized the scrutinized lead compounds for protein ligand docking. Screened lead compounds were further investigated for molecular docking studies. STRING server was employed to explore protein-protein interactions of TNFRSF10B target protein. TNFSF10 protein showed highest 0.999 confidence score and selected protein-protein docking by utilizing GRAMM-X server. *In-silico* docking results revealed I-58, S-90 and A-62 as most active interacting residues of TNFRSF10B receptor protein with R-130, S-156 and R-130 of TNFSF10B ligand protein.

**Conclusion:**

Current research may provide a backbone for understanding structural and functional insights of TNFRSF10B protein. The designed novel inhibitors and predicted interactions might serve to inhibit the disease. Effective *in-vitro* potent ligands are required which will be helpful in future to design a drug to against Head and neck cancer disease. There is an urgent need for affective drug designing of head and neck cancer and computational tools for examining candidate genes more efficiently and accurately are required.

## Background

HNC is the sixth most occurring cancers worldwide [1] whereas in Pakistan, the second most prevalent cancer affecting the pharynx, larynx and oral cavity [2]. Multigenic nature and environmental agents made heterogeneous and complex epidemiology of disease [3]. Different genetic polymorphisms are reported in enzymes involved in the metabolization of alcohol and tobacco greatly increases the risk of Squamous Cell Carcinoma of Head and Neck cancer (SCCHN) [4,5]. Morbidity and prognosis differ from patient to patient depending on causative agents, anatomical site and the stage of disease.

DNA modifications and structural variations in the genomic content of cell controlling gene expression are responsible for cancers. Deletions and duplications of chromosomal segments or even whole chromosome lead to the genomic instability causing genetic alterations [6]. DNA modifications are greatly responsible for change in expression level of HNC [6,7]. Genetic events result in the activation of proto-oncogenes and inactivation of tumor suppressor genes or both, leading to the development of SCCHN [7,8].

Tumor Necrosis Factor (TNF) is a mediator pro-inflammatory cytokine involved in the progression and development of cancer. The family of TNF inhibits tumor formation through apoptosis but TNFs deregulation encourages metastasis, migration and invasion of tumor cell tissues [9]. TNFRSF10B gene consists of 10 gene coding exons. Sequence analysis of all exons suggested allelic loss of 8p was in 20 primary HNCs. A number of putative tumors suppressor genes are located on 8p region which is a frequent site of translocations in head and neck tumors. In 1998, 2-bp insertion in this gene at residue 1065 was found that introduces a premature stop codon lead to truncated protein in SCCHN. Sequence comparison between patient and normal tissues confirmed that germ line contains truncating mutation in the absence of p53 mutation [10].

*In-silico* analysis of TNFRSF10B gene was conducted to elucidate the novel molecules, interacting partners, their binding interactions and to find a most plausible functions. The main objective of our study was to design novel inhibitors. The aim of research was to elucidate the interactions of TNFRSF10B protein with novel inhibitors and to identify the relation of gene with disease.

## Results

The current work presents bioinformatics analysis of TNFRSF10B, a candidate gene of HNC. TNFRSF10B gene mapped on chromosome 8, started from 22877646 bp and ends with 22926692 bp. Molecular functions, biological processes and cellular locations of TNFRSF10B gene are mentioned in Table 1.

**Table 1:** Molecular functions, biological processes and cellular locations of TNFRSF10B gene

**Gene**	**Molecular function**	**Biological process**	**Cellular location**
		Apoptotic process	
	Receptor activity		Plasma membrane
		Extrinsic apoptotic signalling pathway via death domain receptors.	
	Protein binding		Integral to membrane
***TNFRSF10B***		Regulation of apoptotic process.	
	TRAIL binding		
		Activation of cysteine-type endopeptidase activity involved in the apoptotic process.	

**Figure 1:**
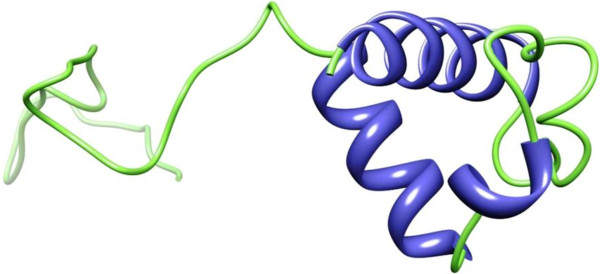
**3D structure of *TNFRSF10B *protein visualized by the UCSF CHIMERA visualizing tool.** A model is presented in smoothing ribbons and sticks. Helixes are represented by blue colour and coils by green colour.

**Figure 2:**
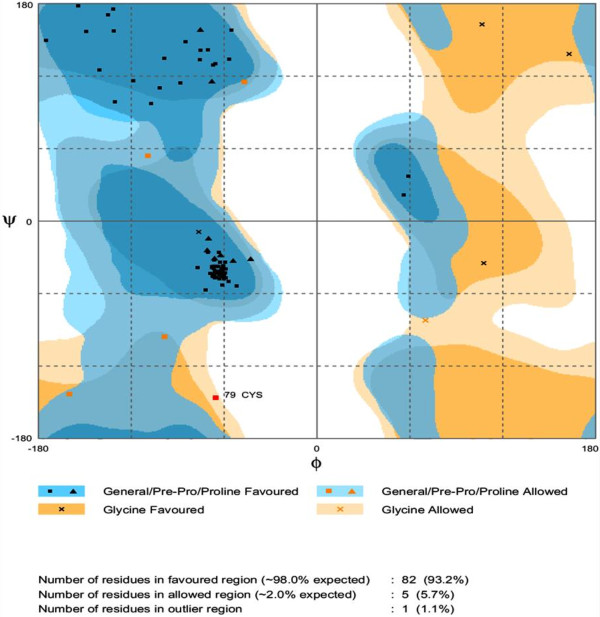
**RAMPAGE result of *TNFRSF10B *protein showing the distribution of residues in favored, allowed and outlier regions.**

**Figure 3:**
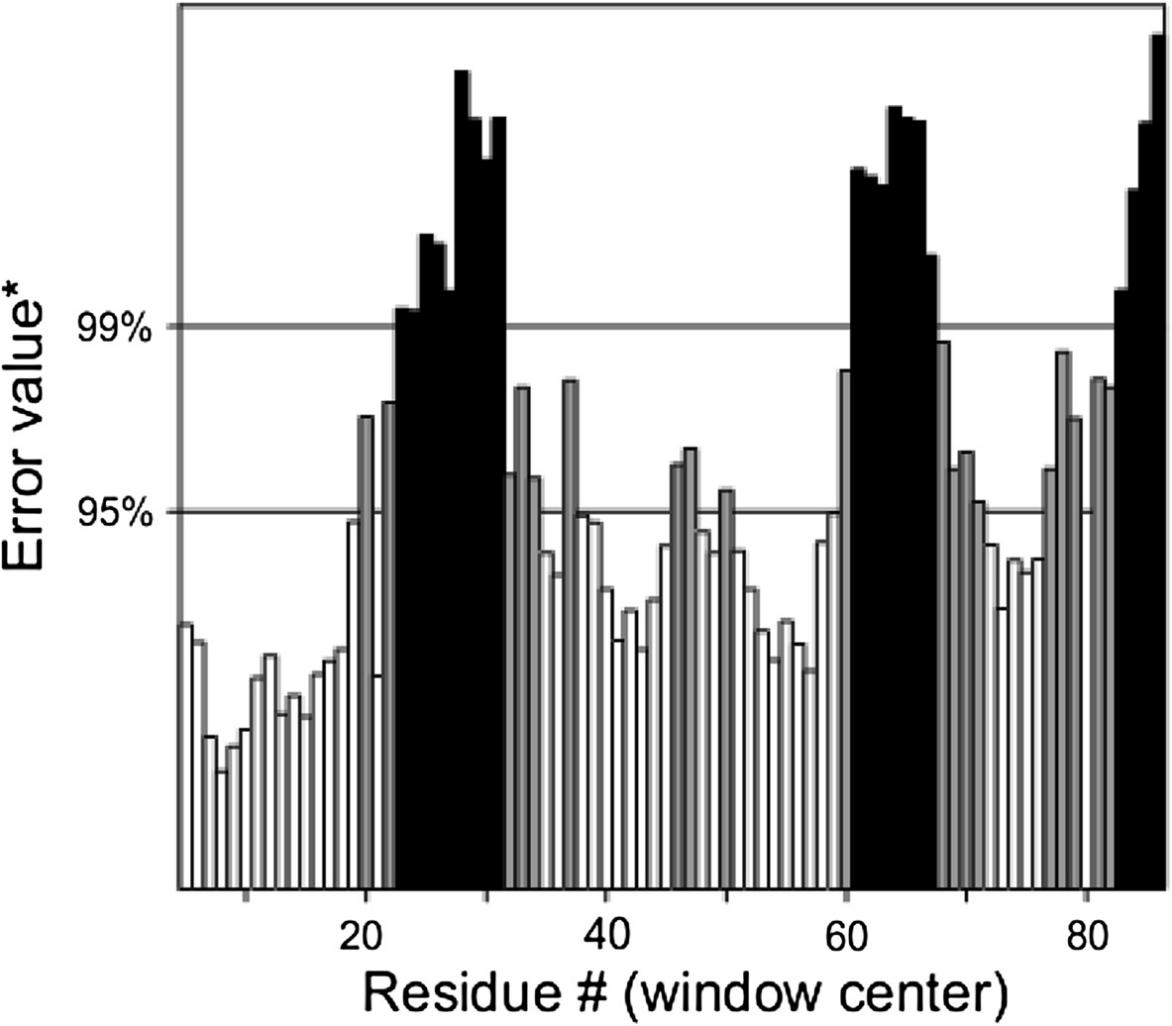
**ERRAT result of *TNFRSF10B *model showing the 51.85% overall quality of model.** X-axis shows the number of resides while the Y-axis represents error values of residues.

**Figure 4:**
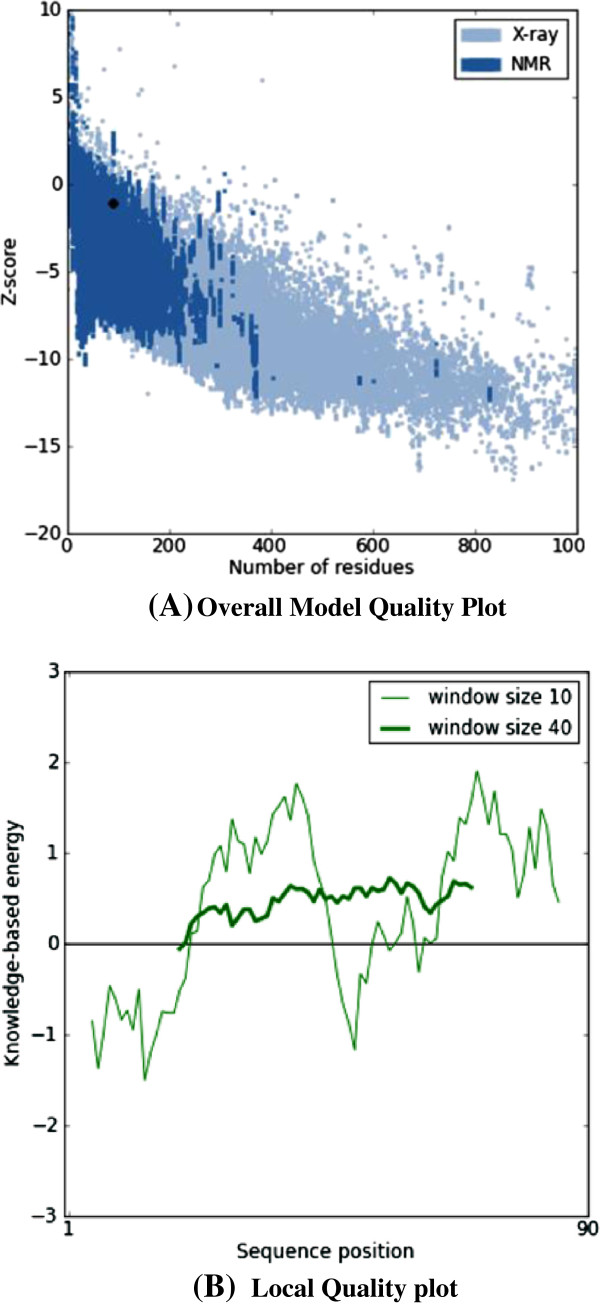
**ProSA results of *TNFRSF10B *model (A) Plot showing −1.08 Z-score representing the overall quality of the model. (B)** Local model quality plot of model showing position of sequences against knowledge based energy in both window sizes 10 and 40.

Protein sequence of TNFRSF10B in FASTA format was retrieved from UniprotKB with accession number E9PBT3. Table 2 represents the templates of *TNFRSF10B* protein selected on overall quality, total score and query coverage. All the three selected templates were used for three dimensional structure predictions by comparative modeling. The best model was built by MODELLER (9v10) [11] by using 2ZB9 template with optimal alignment. Predicted model was visualized by Chimera 1.6 [12] shown in Figure 1. Assessment of predicted structure by Rampage, ERRAT and ProSA is shown from Figures 2, 3 and 4 respectively.

**Table 2:** Template aligned by high score and query coverage

**Accession ID**	**Total score**	**Query coverage**	**E-value**	**Sequence identity**
**2ZB9**	25.8	84%	5.4	30%
**3NKE**	25.8	46%	4.1	32%
**3NKD**	25.0	46%	8.1	32%

**Figure 5:**
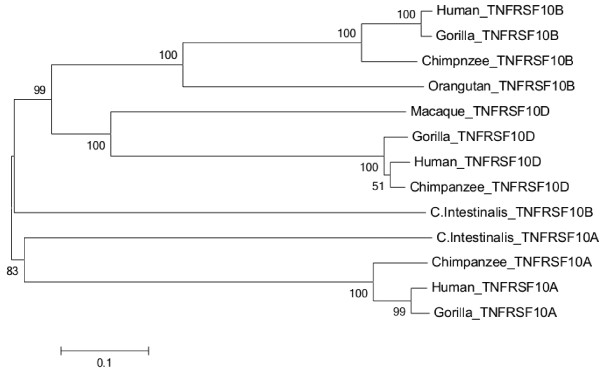
**Neighbor-joining tree of *TNFRSF10B *gene showing *TNFRSF10A *gene as ancestral gene.** Topology of tree showing *TNFRSF10A* gene is out group of tree. Species having >50% bootstrap values are presented in this tree.

The evolutionary tool MEGA 5 [13] was employed to construct a neighbor-joining tree of TNFRSF10B gene. Ensembl BLAST (http://www.ensembl.org/Multi/blastview) was performed to identify paralogs of the target gene. Protein sequences of TNFRSF10A, TNFRSF10D and TNFRSF10B were retrieved to determine the evolutionary relationship between paralogs and orthologs. Numbers of bootstrap replications were 1000 in bootstrap method. P-distance method and complete deletion option were used in the construction of neighbor-joining tree shown in Figure 5.

### Virtual screening technique

**Figure 6:**
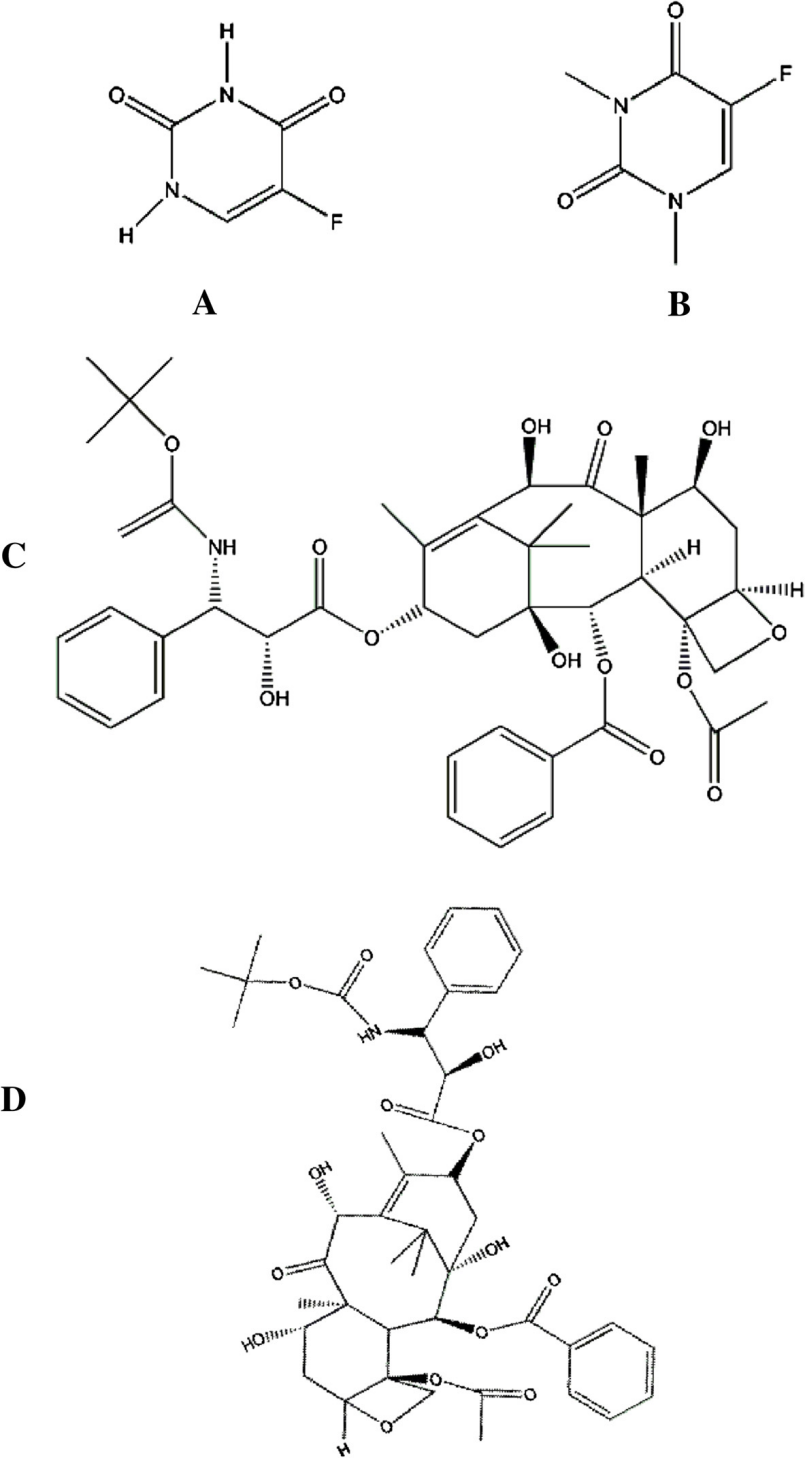
Chemical structures of screened lead compounds (A, B, C and D) used in docking analysis.

Virtual screening approach was employed to identify competitive compounds that inhibit the mutated TNFRSF10B activity. In pharmaceutical industry, the approach has become progressively more popular for lead identification. The main objective of virtual screening is to screen a large set of compounds against specific receptor protein to identify the manageable number of inhibitors for possibly chance of lead to drug candidate [14]. Four lead compounds (A, B, C and D) structures were screened for further analysis shown in Figure 6.

Bioavailability and membrane permeability are the molecular properties that always connected with molecular weight, partition coefficient (logP), number of hydrogen bond donors and number of H-bond acceptors as a basic molecular descriptors [15]. Lipinski “Rule of Five” was formulated by using these molecular properties [16]. According to this rule, molecules with good membrane permeability have log P≤5, molecular weight ≤500, hydrogen bond acceptors ≤10 and donors ≤5 [17]. Therefore, Lipinski’s Rule of Five was applied to check the bioavailability characteristics such as absorption, distribution, metabolism, elimination (ADME) of the lead compounds. In present work, these properties were determined by Mcule tool [18] mentioned in Table 3.

**Table 3:** Drug related properties of the designed molecules

**Inhibitors**	**Molecular mass**	**LogP**	**Rotatable bonds**	**H-bond donors**	**H-bond acceptor**	**RoF violation**	**Interacting residues**
**A**	130.077	−0.7977	0	2	4	0	ARG-23, GLU-24, ALA-25, ARG-26, GLY-27, ALA-28, VAL-39, VAL-41, LEU-46
**B**	158.303	−0.776	0	0	4	0	ARG-23, GLU-24, VAL-39, LEU-40, VAL-41, ALA-43, LEU-46
**C**	789.86	3.5317	14	5	14	2	ILE-58, ALA-59, SER-60, ALA-62, MET-73, ILE-85, GLN-86, TRP-89, SER-90
**D**	807.877	3.65.05	14	5	15	2	PRO-9, ALA-10, SER-12, GLY-13, LYS-16, ARG-17 PRO-30, GLN-53, LYS-54, GLU-57

### Toxicity

High quality lead structures are the requirement for the successful drug discovery and structures of drug properties are more acceptable than common [19]. In the early steps of drug discovery, poor pharmacokinetics and toxicity should be eradicated. Toxicity and drug score characteristics were further used to screen the hits [20].

**Figure 7:**
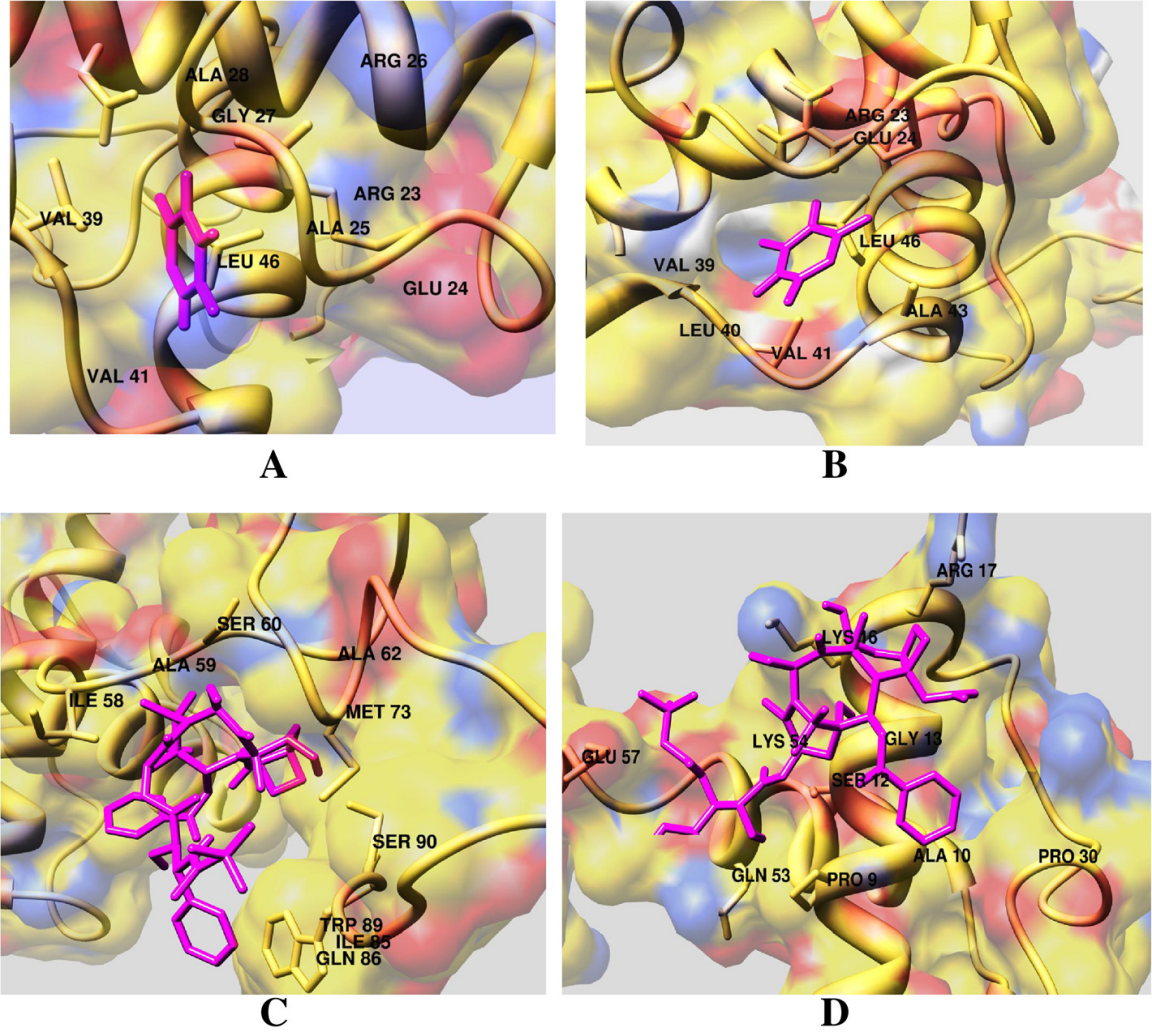
**Binding views of lead compounds (A, B, C and D) with TNFRSF10B receptor protein.** The compounds are depicted in pink sticks while protein structures in brown hetromeric surfaces. Interacting residues of receptor protein docked with respective four (**A**, **B**, **C** and **D**) compounds are determined and presented by Chimera.

**Figure 8:**
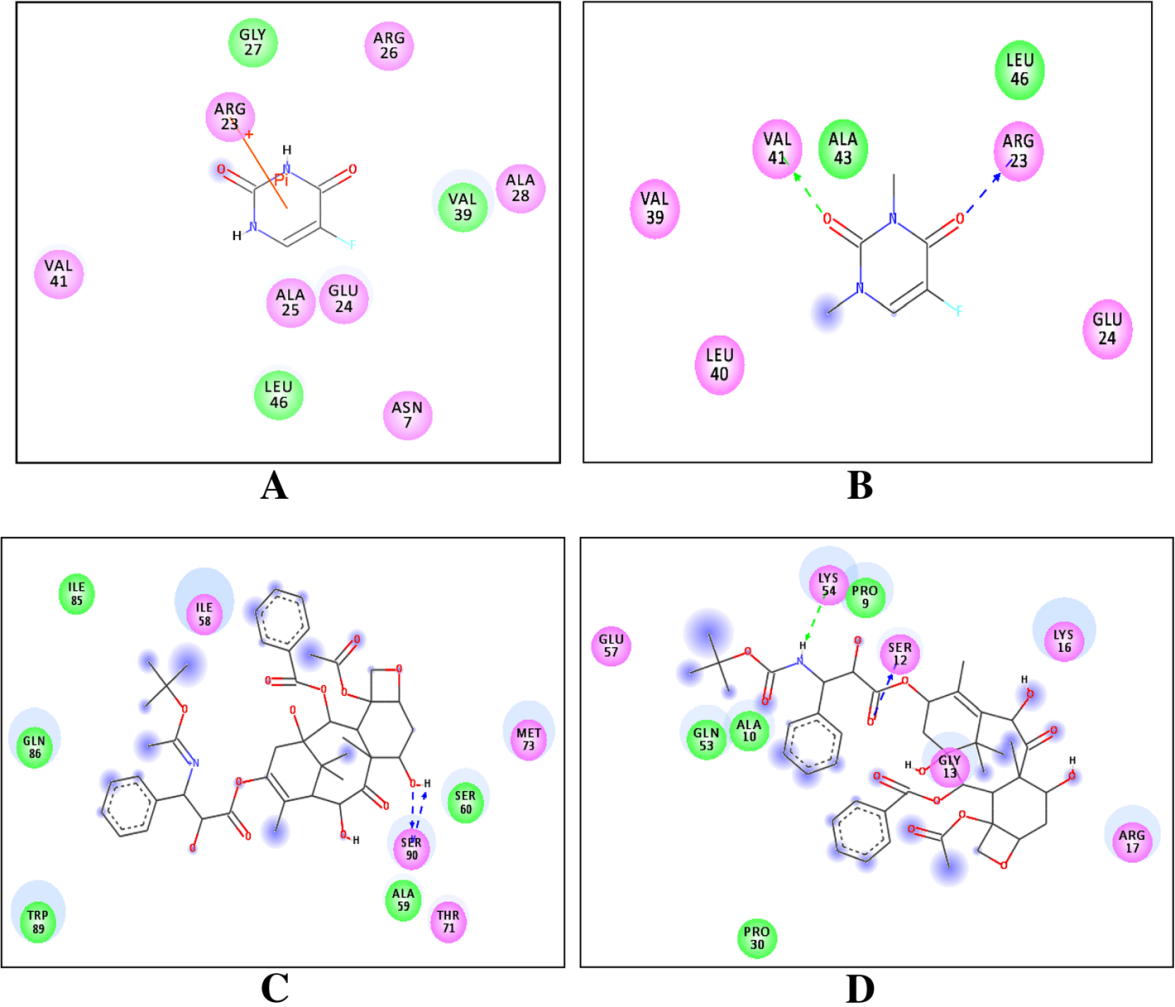
**Post dock analysis of complexes by Discovery Studio tool.** Chemical structures of compounds with interactions of receptor protein are shown. **A**) Interactions of novel designed compound A with receptor protein. **B**) Compound B interacted with TNFRSF10B. **C**) Interacted complex of TNFRSF10B with designed C inhibitor. **D**) Binding residues of compound D with receptor protein.

Docking analysis of TNFRSF10B protein with screened lead compounds was performed by AutoDock and post dock analysis by Chimera 1.6v and Discovery Studio. The amino acids present in the active sites of the protein were identified by observing those amino acids in the vicinity of 4 Å. Residues of receptor protein interacting with compounds were calculated and presented in Figures 7 and 8 by Chimera and Discovery Studio respectively.

**Figure 9:**
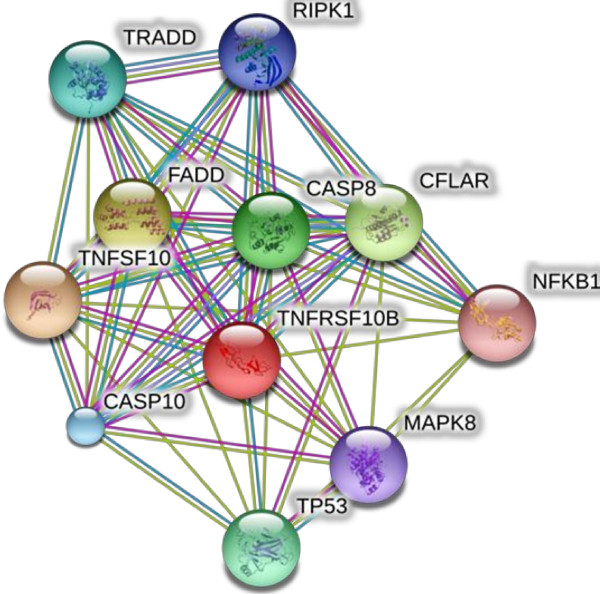
**Interaction network of *TNFRSF10B *generated by STRING database.** In this network *TNFSF10B* protein showed the highest interaction score 0.999 with *TNFSF10* protein. *TNFSF10* protein is used in protein-protein docking of *TNFRSF10B*.

**Figure 10:**
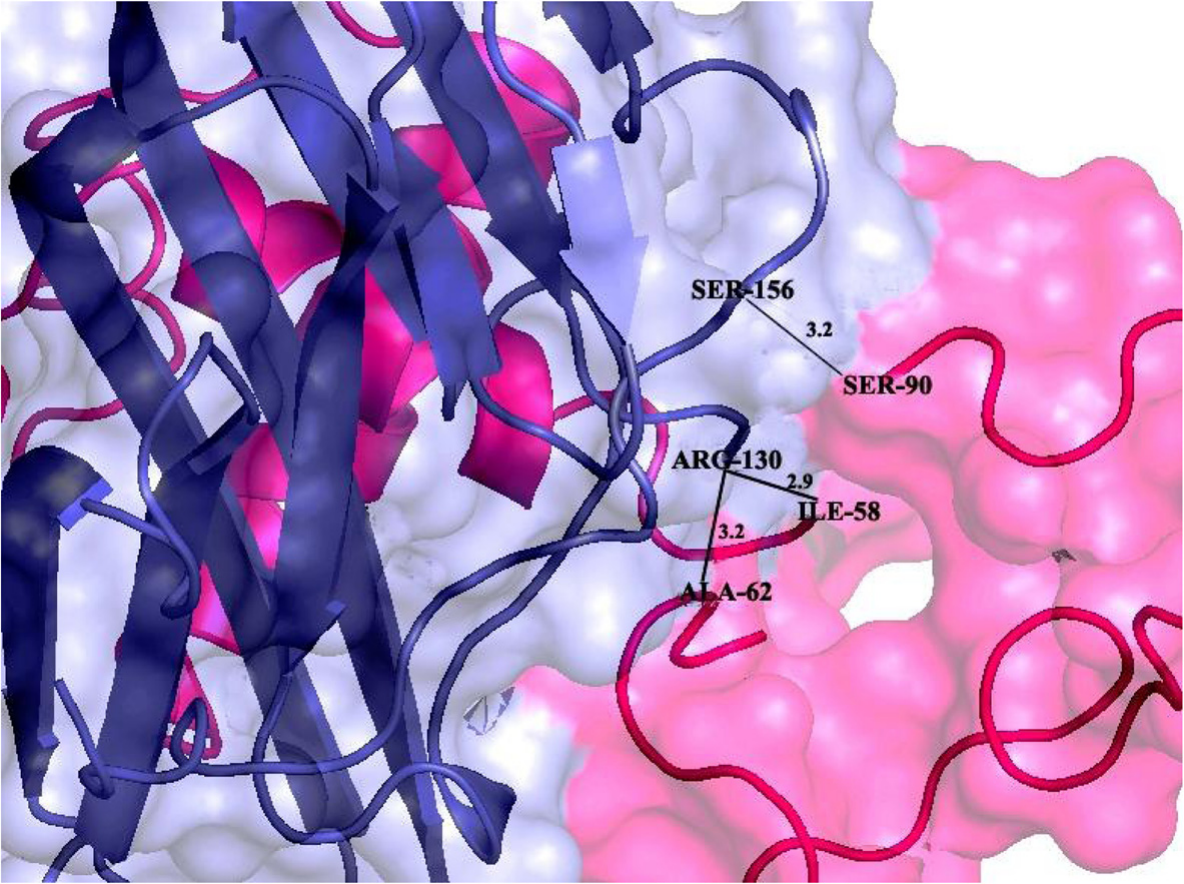
**Interaction analysis of *TNFRSF10B *and *TNFSF10 *visualized by PyMol tool.**

A functional partner network of TNFRSF10B protein was generated by the STRING [21] and STITCH3 [22] databases to explore the highly interacting proteins of the target protein. TNFSF10 protein having highest interaction score 0.999 with receptor protein was used as a ligand protein in protein-protein docking by GRAMM-X [23] and Hex [24]. Interaction network and protein-protein docked complex are shown in Figures 9 and 10 respectively. Interactions of interacting residues were determined and analyzed by PyMol mentioned in Table 4.

**Table 4:** **Interacting residues between *TNFRSF10B *and *TNFSF10 *proteins**

**Receptor protein**	**Interacting protein**	**Interactions type**	**Interactions (Receptor residue → Interacting protein residue**	**Bond distance (Å)**
***TNFRSF10B***	*TNFSF10*	Ionic bonding (N-O)	ILE-58/O **→** ARG-130/NH1	2.9
			SER-90/O → SER-156/N	3.2
			ALA-62/N → ARG-130/O	3.2

## Discussion

Head and neck cancer remains a disfiguring disease associated with a high mortality rate [25]. Progressive accumulation of genetic aberrations leads to SCCHN but exact nature is still unknown. Candidate gene identification approach may provide key factors to pinpoint candidate genes involved in different carcinomas, which leads to explore the receptor-ligand or protein-protein interactions recognize these carcinomas that might lead to the development of effective therapeutic strategies [26].

For head and neck candidate gene TNFRSF10B, 2ZB9, 3NKE and 3NKD templates were retrieved from PDB. Out of these three templates, 2ZB9 showed optimal alignment and query coverage. Rampage showed the 93.2% residues in favored region and 5.7% in the allowing region whereas only 1 residue was in outlier region. 51.852% quality factor and −1.08 Z-score were shown by ERRAT and ProSA evaluation tools respectively.

In literature, TNFRSF10B gene and its paralogs genes are predicted in primates and human. MEGA 5 was employed using neighbor-joining method to determine evolutionary relationship of genes among teleosts, rodents, birds, primates and mammals. TNFRSF10D and TNFRSF10A are the paralogs of TNFRSF10B gene that are used in the construction of a phylogenetic tree. TNFRSF10A gene is outgroup in TNFRSF10B tree that presents TNFRSF10A gene as an origin of other genes. TNFRSF10A gene gave rise to TNFRSF10B gene in *Ciona Intestinalis* and other cluster is further diverged into TNFRSF10D and TNFRSF10B genes. Human and gorilla are closely related in TNFRSF10B and TNFRSF10A genes while in TNFRSF10D, human is closely related with chimpanzee. Bootstrap replication values >50 are presented in phylogenetic tree representing the reliability of topology.

Novel designed molecules have drug related properties and served as inhibitors for candidate gene. Interactions were observed in binding pocket of TNFRSF10B showing polar nature of binding domain. The designed compounds fulfill the properties of a competent drug and have no toxicity, mutagenic, irritants and carcinogenic property.

TNFSF10 protein showed highest interacting score 0.999 with TNFRSF10B target protein belonging to the same protein family. TNFSF10 protein was used as a ligand for protein-protein docking with TNFRSF10B receptor protein. Post docking analysis was performed by PyMol to analyze the hydrogen, ionic and hydrophobic interactions. Only ionic interactions were found in docked complex. Isoleucine-58 of receptor protein TNFRSF10B showed ionic interactions with Arginine-130 of ligand protein TNFSF10 with the distance of 2.9 Å. The nitrogen atom of arginine showed interaction with oxygen atom of isoleucine. Serine-90 of TNFRSF10B receptor protein showed ionic interactions with Serine-156 of ligand protein with a bond distance of 3.2 Å. Nitrogen of serine of ligand protein TNFSF10 interacted with the oxygen of serine of receptor protein. Alanine-62 nitrogen of receptor protein TNFRSF10B interacted with arginine-130 oxygen of ligand protein TNFSF10 with 3.2 Å bond distance.

## Conclusion

For protein-protein interaction and novel designed molecules, both functional and expressional studies of TNFRSF10B showed significant interaction. *In-vivo* experimentation could be performed in animal model to check the effect of selected protein interactions which may leads to the approved drug of head and neck cancer. More than 80% homology between human and primates are strong evidence to build ancestral relationship which will help in prediction of protein functions and family. Current research suggested a baseline for novel ligand screening, docking and ancestral hierarchy for development and validation of novel drugs in particular function prediction of candidate gene TNFRSF10B.

## Materials and methods

The sequence of TNFRSF10B protein in FASTA format was retrieved from Uniprot Knowledge base (http://www.uniprot.org/) of accession number E9PBT3. The retrieved amino acid sequence of TNFRSF10B was subjected to a protein-protein BLAST (BLASTp) search against the Protein Data Bank (PDB) (http://www.rcsb.org/) [27] to recognize a suitable template structure. Suitable template [PDB ID: 2ZB9] having 84% query coverage, 30% sequence identity and 5.1 E-value was used in comparative modeling of TNFRSF10B protein. The homology modeling program MODELLER 9v10 was applied to generate 3D models. TNFRSF10B 3D model having lowest objective function was further assessed by Rampage [28], ERRAT [29] and ProSA [30] evaluation tools for the reliability of predicted structure.

Molecular Evolutionary Genetic Algorithm (MEGA 5) was used to infer ancestral history and species relationship of TNFRSF10B gene. Distance based approach through neighbor-joining technique was applied to construct the phylogenetic tree by using 1000 bootstrap replicates.

The compounds obtained through virtual screening were used in docking analysis by AutoDock Vina [31]. The ligand molecules of target protein were not reported in earlier studies and also not found in biological databases, hence virtual screening technique was used to screen drug like lead compounds for TNFRSF10B docking calculations. Four novel lead compounds (as A, B, C and D) were screened. Mcule suit was employed for virtual screening and to predict the bioactivity and molecular properties of lead compounds.

LogP value is an important predictor of per oral bioavailability of drug molecules [32]. Therefore, physiochemical properties such as LogP, molecular mass, rotatable bonds, hydrogen bond acceptor and donors of 4 selected lead compounds were determined. Results showed that compounds (C and D) showed violations of two rules of lipinski rule of five suggesting that lead compounds have good bioavailability. The selected top scrutinized compounds were minimized through VegaZZ [33] and ChemDraw Ultra [34]. Subsequent analysis on selected lead compounds were carried out and docking analysis was performed to identify the binding affinities by AutoDock. Parameters of AutoDock used in docking are mentioned in Table 5.

**Table 5:** Parameters of AutoDock used in docking analysis

**Lead compounds**	**Centre**			Size (X-axis* Y-axis* Z-axis)	**Rate of gene mutation**	**Rate of crossover**	**Binding affinity (Kcal/mol)**
	**X-axis**	**Y-axis**	**Z-axis**				
**A**	5.52	61.778	35.2	40*40*40	0.02	0.8	−4.8
**B**	5.846	61.933	35.423	40*40*40	0.02	0.8	−4.9
**C**	5.846	61.933	35.423	40*40*40	0.02	0.8	−8.6
**D**	5.846	61.933	35.423	40*40*40	0.02	0.8	−9.9

The STRING and STITCH3 servers were employed to identify the functional partners of TNFRSF10B. These databases are online database of known and predicted protein interactions including direct (physical) and indirect (functional) relationships. Gramm-X and Hex online servers were applied in protein docking of TNFRSF10B protein with its interactive partner TNFSF10 protein. Post docking analysis of docked complex was performed by PyMol tool.

## Competing interests

The authors declare that they have no competing interests.

## Authors’ contributions

RAT and SAS has equal contribution. RAT and SAS carried out analysis and drafted the manuscript under the supervision of NAK. AM and NAK defined the research theme, designed methods, and analyzed the data. JZKK, AM and NAK critically studied the manuscript. All authors have contributed to, seen, read and approved the manuscript.
